# SvSXP: a *Strongylus vulgaris* antigen with potential for prepatent diagnosis

**DOI:** 10.1186/1756-3305-6-84

**Published:** 2013-04-04

**Authors:** Ulla V Andersen, Daniel K Howe, Sriveny Dangoudoubiyam, Nils Toft, Craig R Reinemeyer, Eugene T Lyons, Susanne N Olsen, Jesper Monrad, Peter Nejsum, Martin K Nielsen

**Affiliations:** 1 Department of Large Animal Science, Faculty of Health and Medical Sciences, University of Copenhagen, Copenhagen, Denmark; 2 M.H. Gluck Equine Research Center, Department of Veterinary Science, University of Kentucky, Lexington, KY, USA; 3 East Tennessee Clinical Research, Inc., Rockwood, TN, USA; 4 Danish Centre for Experimental Parasitology, Department of Veterinary Disease Biology, Faculty of Health Science, University of Copenhagen, Frederiksberg, Denmark

**Keywords:** SXP, IgG(T), *Strongylus vulgaris*, Prepatent, Diagnosis, cDNA library, ELISA, Validation

## Abstract

**Background:**

Strongyle parasites are ubiquitous in grazing horses. *Strongylus vulgaris*, the most pathogenic of the large strongyles, is known for its extensive migration in the mesenteric arterial system. The lifecycle of *S. vulgaris* is characterised by a long prepatent period where the migrating larvae are virtually undetectable as there currently is no test available for diagnosing prepatent *S. vulgaris* infection. Presence of *S. vulgaris* larvae in the arterial system causes endarteritis and thrombosis with a risk of non-strangulating intestinal infarctions. Emergence of anthelmintic resistance among cyathostomins has led to recommendations of reduced treatment intensity by targeting horses that exceed a predetermined strongyle faecal egg count threshold. One study suggests an apparent increase in prevalence of *S. vulgaris* on farms where reduced anthelmintic treatment intensity has been implemented. These issues highlight the need for an accurate and reliable assay for diagnosing prepatent *S. vulgaris* infection.

**Methods:**

Immunoscreening of a larval *S. vulgaris* cDNA library using hyperimmune serum raised against *S. vulgaris* excretory/secretory antigens was performed to identify potential diagnostic antigens. Immunoreactive clones were sequenced, one potential antigen was characterised, expressed as a recombinant protein, initially evaluated by western blot (WB) analysis, the diagnostic potential of the IgG subclasses was evaluated by ELISA, and the diagnostic accuracy evaluated using serum from 102 horses with known *S. vulgaris* infection status.

**Results:**

The clone expressing the potential antigen encoded a *S. vulgaris* SXP/RAL2 homologue. The recombinant protein, rSvSXP, was shown to be a potential diagnostic antigen by WB analysis, and a target of serum IgGa, IgG(T) and total IgG in naturally infected horses, with IgG(T) antibodies being the most reliable indicator of *S. vulgaris* infection in horses. Evaluation of diagnostic accuracy of the ELISA resulted in a sensitivity of 73.3%, a specificity of 81.0%, a diagnostic odds ratio of 11.69; a positive likelihood ratio (LR) of 3.85 and a negative LR was 0.33. The area under the ROC curve was 0.820.

**Conclusion:**

IgG(T) antibodies to recombinant SvSXP show potential for use as an antigen for prepatent diagnosis of migrating stages of *S. vulgaris* with moderate to good diagnostic accuracy.

## Background

Strongyle parasites are ubiquitous in grazing horses with more than 50 different species described [1]. The most pathogenic of the equine gastrointestinal parasites is *Strongylus vulgaris*. The prepatent period of *S. vulgaris* is 6–7 months [2], and during this time, the larvae migrate in the Cranial Mesenteric Artery (CMA) and major branches [3,4]. Here, the larvae cause verminous endarteritis [5-7], and subsequent thromboembolism can cause a painful non-strangulating infarction of the intestinal tract [3,8].

Prior to the advent of modern paste-based dewormers, *S. vulgaris* was present in about 80–100% of horses [9,10], but frequent interval-dose anthelmintic regimens appear to have caused a dramatic reduction in prevalence [11,12]. However, these frequent treatments have led to anthelmintic resistance in other parasite categories infecting horses; cyathostomins [13-15] and *Parascaris equorum*[16-18], and recommendations are now given to reduce treatment intensity by basing control programs on systematic parasite surveillance [19-21]. In the European Union, prescription-only restrictions on anthelmintic drugs have been or are being implemented by legislation of member countries [22]. Experiences with prescription-only restrictions in Denmark show that a reduction in treatment intensity follows [23]. A recent study performed in this country suggested that these anthelmintic treatment policies may have contributed to a higher prevalence of *S. vulgaris*[24]. Given the high pathogenicity of this parasite, there is a need for improved diagnostic assays to better diagnose and monitor this parasite while maintaining anthelmintic efficacy in equine populations.

Currently, diagnosis of *S. vulgaris* infection is based on the presence of eggs shed in faeces of infected horses, and is accomplished by either larval culture and subsequent microscopic examination [25,26] or by a semi-quantitative PCR detecting DNA extracted from the eggs [27]. So far, no test has been developed to accurately diagnose the presence of migrating larvae in the CMA and branches [Reviewed by [28]].

Several attempts have been made to develop a serological test for the diagnosis of prepatent *S. vulgaris* infection. In the last three decades, whole worm extracts, surface antigen extracts and excretory/secretory (ES) antigens have been evaluated for use in diagnostic assays [29-33]. Wynne and co-workers [29] evaluated different tissue extracts and ES antigens by use of hyperimmune rabbit sera raised against the different antigenic fractions. This led to the discovery of two species-specific and one stage-specific ES antigen, but these were not evaluated with serum from horses naturally or experimentally infected with *S. vulgaris*. Thus the full diagnostic potential of these antigens remains unknown. Klei *et al.*[30] evaluated the unspecific antibody response in previously helminth-naïve foals that were experimentally infected with *S. vulgaris*. These workers developed an indirect fluorescent antibody (IFA) assay and discovered that these foals developed a species-specific response to *S. vulgaris* L_3_-larvae with no cross-reactivity with *S. edentatus*, *S. equinus*, or cyathostomins. However, evaluation of hyperimmune rabbit sera raised against *S. edentatus* and *S. equinus* antigens showed that these cross-reacted with *S. vulgaris* larvae; in fact both sera reacted more strongly against *S. vulgaris* larvae than *S. edentatus* or *S. equinus* larvae. Therefore, the IFA was never validated as a diagnostic test. Nichol and Masterson [31] evaluated *S. vulgaris* surface antigen extracts and found them to show a high degree of cross-reactivity with the closely related *S. edentatus* and the more distantly related *Parascaris equorum*. Affinity chromatography was performed to remove cross-reactive antibodies from the hyperimmune rabbit serum and three antigens were found to be potential diagnostic antigens. Adeyefa [32] evaluated somatic extracts using the IgG(T) fraction of serum from horses naturally infected with gastrointestinal helminths including arterial *S. vulgaris* larvae and found two potential diagnostic antigens. Cross-reactivity with other gastrointestinal helminths was, however, not assessed. Hassan *et al.*[33] recently evaluated adult somatic antigen and found the ELISA to detect more positive samples than the larval culture. No horses were necropsied for determination of true infection status, and ELISA results were only compared to larval cultures, so diagnostic performance cannot be assessed [33]. Taken together, none of the potential antigens described here were fully evaluated or validated as diagnostic tests, and most of them were not fully characterized. The antigens discovered by Nichol and Masterson [31] were the only antigens to be isolated and expressed *in vitro*[31], but sera from naturally infected horses failed to recognise these. No attempt has been made to clone the *S. vulgaris* antigen or express recombinantly for incorporation into a diagnostic test.

Recently, a molecular approach was employed for identifying candidate molecules for prepatent diagnosis of another important parasite group infecting horses; larval cyathostomins. This included immunoscreening of a cDNA library constructed from encysted cyathostomin larvae and allowed identification of a promising antigen to be evaluated as a candidate for diagnosing encysted cyathostomin larvae [34]. This protein was found to be stage-specific as it is only expressed in the larval stages of the cyathostomins.

This study employed immunoscreening of a *S. vulgaris* larval cDNA library to identify genes that encode potential diagnostic antigens. The aims were to subsequently explore the use of these in immunodiagnostic assays for a diagnosis of prepatent *S. vulgaris* infection, to evaluate the inter- and intra-assay variability, the diagnostic properties, as well as the quantitative aspects of the assay.

## Methods

### Horses

A total of 102 horses with necropsy-confirmed status of *S. vulgaris* infection were enrolled in the validation study. All necropsies were performed at either University of Kentucky in Lexington, Kentucky or East Tennessee Clinical Research (ETCR) in Rockwood, Tennessee.

All horses from University of Kentucky were naturally infected with mixed species of gastrointestinal helminth infections (n=31). They were enrolled from two main populations; a herd kept without anthelmintic intervention since 1979 [35], and a population of research horses maintained with four anthelmintic treatments a year. Naturally infected horses from Tennessee (*n*=23) participated in different anthelmintic drug trials at ETCR and all underwent necropsy. In total, samples from 54 naturally infected horses were collected.

Experimental infections were carried out for two different research projects with horses maintained at ECTR (n=48). Horses in group P (*n*=20) were treated once daily for five consecutive days with fenbendazole paste (10 mg/kg, Panacur, Merck, Summit, NJ, USA), housed in stables and inoculated on a single occasion with 600 embryonated *P. equorum* eggs obtained locally from naturally infected horses. After six months the horses were euthanatised and necropsied. Horses in group S (*n*=28) were acquired with unknown anthelmintic treatment history, and were therefore treated with a larvicidal regimen of moxidectin (0.4 μg/kg, Quest gel, Pfizer, Madison, NJ, USA) administered once, and fenbendazole paste (10 mg/kg, Panacur) once daily for five consecutive days during the week prior to enrolment in the study. They were subsequently infected with 5,000 cyathostomin third stage larvae (L_3_) five times weekly throughout the study. Five weeks into the study the horses started receiving 25 *S. vulgaris* L_3_ larvae once weekly until euthanasia and necropsy after 5.5 months [36].

At necropsy, the posterior aorta and cranial mesenteric artery and branches were recovered and evaluated for the presence of migrating *S. vulgaris* larvae in all horses.

The case-definition that served as the gold standard for classification of *S. vulgaris* positive horses was: horses with migratory tracts, one or more larvae, or evidence of previous infection. Migratory tracts were considered evidence of a current infection, and a circular area with raised surface, roughened or corrugated intima without evidence of active thrombosis was classified as evidence of previous infection. Horses with no migratory tracts, no larvae, and no signs of previous infection were classified as being *S. vulgaris*-negative.

The gastrointestinal and migrating parasites were enumerated as previously described [37].

A peripheral blood sample was collected from each horse (*n*=102) in a serum or serum-separator tube. Sera were separated by centrifugation, and duplicate aliquots were transferred to 2 mL cryovials and stored at −20°C until analysis. The serum was stored for up to 5 years prior to analysis.

The horses in this study had a mean age of 19.5 months (range 0.6 months – 22 years) with a median of 12 months of age. A total of 46 horses were female and 54 were males of which 12 were castrated. Data on age and gender was missing for two and one horse, respectively. The subset of horses 7 months and older had a mean age of 22.51 months (range 7 months – 22 years) with a median of 18 months of age with 44 female, 42 males of which 12 were castrated. Data from horses 3 months and younger were omitted; the next youngest foal was 7 months old. Breeds represented included: Tennessee Walking Horse, Paint, American Quarter Horse, Thoroughbred, Standardbred, Appaloosa, Shetland type ponies and mixed light breed.

All work involving the horses at UK and ETCR was approved by the Institutional Animal Care and Use Committee.

### Parasite material

Migrating *S. vulgaris* larvae were collected by dissection of the abdominal aorta, celiac artery, cranial mesenteric artery and major branches recovered from horses at the University of Kentucky that were naturally infected with gastrointestinal parasites and where anthelmintic drugs have not been used since 1979 [35]. The larvae were carefully lifted from the thrombus material and washed four times in 20 mL PBS (137 mM NaCl, 10 mM phosphate, 2.7 mM KCl, pH 7.4) to remove debris. The larvae were either placed in 2 mL cryotubes and snap frozen in liquid N_2_ for RNA extraction or used for collection of ES antigens.

Worms were collected from the caeca of horses using the necropsy technique previously described [37], adult *S. vulgaris* worms were identified by morphological criteria [1] and washed five times in 20 mL PBS to remove debris and used for collection of ES antigens.

### Excretory/secretory antigens

Living washed adult worms were incubated in 5 mL RPMI-1640 (Life Technologies, Grand Island, NY, USA) with penicillin (100 IU/mL), streptomycin (100 IU/mL), amphotericin B (0.25 μg/mL) and a protease inhibitor cocktail (Protease inhibitor cocktail, P2714, Sigma-Aldrich, St. Louis, MO, USA) in a 5% CO_2_ incubator at 37°C. The medium was collected after 12 and 24 h and fresh medium was added. The protein concentration was analysed using the Bradford Protein quantitation assay (Pierce, Rockford, IL, USA) as per the manufacturer’s protocol. The ES antigen-rich medium was then dialysed against PBS at 4°C using a 3 mL 3.5 kDa molecular cut-off Slide-A-Lyzer® (Pierce, Rockford, IL, USA) according to the manufacturer’s protocol. Dialysed ES antigen was frozen and shipped to Cocalico Biologicals, Inc. (Reamstown, PA, USA) for production of hyperimmune serum. One rat was immunised with 250 μg adult *S. vulgaris* ES antigen mixed with Titermax® as adjuvant and given as six inoculations over a 61-day period. Due to background reactivity against *E. coli* proteins, the hyperimmune rat serum was pre-absorbed extensively against *E. coli* XL1-BLUE lysates [38].

Larval ES antigens were obtained from *S. vulgaris* larvae using 2 mL of RPMI-1640 (Life Technologies, Grand Island, NY, USA) with penicillin (100 IU/mL), streptomycin (100 IU/mL), amphotericin B (0.25 μg/mL) and a protease inhibitor cocktail (Protease inhibitor cocktail, P2714, Sigma-Aldrich, St. Louis, MO, USA) and incubating the larvae in a 5% CO_2_ incubator at 37°C. The medium was collected after 14 days during which time the dead larvae were removed daily with a sterile needle.

A total of 300 ng of *S. vulgaris* larval ES antigens was adjusted to 15 μL with water and mixed with 3 μL 5X sodium dodecyl sulphate-polyacrylamide gel electrophoresis (SDS-PAGE) sample buffer containing protease inhibitor cocktail. The same amount of *S. vulgaris* adult ES was prepared similarly. The ES mixtures were denatured at 95°C for 5 min, placed on ice for 5 min and resolved in individual wells under reducing conditions in a 12% polyacrylamide gel, where after the gel was stained by silver staining.

### Construction and immunoscreening of a larval *S. vulgaris* cDNA library

RNA was extracted from N_2_ frozen *S. vulgaris* larvae using the Trizol reagent (Life Technologies, Grand Island, NY, USA) and poly(A)+ RNA (mRNA) was purified from total RNA using the NucleoTrap® mRNA-kit (Clontech, Mountain View, CA, USA) according to the manufacturer's protocol. A total of 400 ng purified mRNA was used as template to synthesise cDNA using the SMART cDNA library construction kit (Clontech, Mountain View, CA, USA), cloned into bacteriophage TriplEx2 lambda vector digested with NdeI and packaged using the Giga Pack III gold packaging extract (Agilent Technologies, Stratagene products division, La Jolla, CA, USA) as previously described for a larval cyathostomin cDNA library [34]. The titre of the cDNA library was evaluated and the quality was assessed by analysing cDNA inserts in 30 randomly-picked plaques by PCR analysis. The PCR products were cleaned using the Wizard® SV Gel and PCR clean-up system (Promega, Madison, WI, USA) and sequenced at the University of Kentucky’s Advanced Genetic Technology Center.

The *S. vulgaris* larval cDNA library was immunoscreened as described by the manufacturer (Clontech, Mountain View, CA, USA). The primary immunoscreening was conducted on 100,000 cDNA clones. Plaque lifts were made onto nitrocellulose membranes (Fisher Scientific, Pittsburg, PA, USA) and the membranes were washed five times for 5 min in 25 mL TBST (100 mM Tris, 0.15 M NaCl and 0.05% Tween-20) and kept in TBST overnight at 4°C. The membranes were blocked with TBST + 1% gelatine for 1 h at room temperature (RT) and probed with preabsorbed hyperimmune rat serum at 1:100 in TBST after washing. As secondary antibodies, horseradish peroxidase (HRP)-conjugated goat anti-rat IgG (H+L) (Jackson ImmunoResearch, Inc. West Grove, PA, USA) were used at 1:10,000 in TBST after washing. The signal was developed using a chromogenic substrate (TMB stabilised substrate for HRP, Promega, Madison, WI, USA) after washing. The membranes were aligned with the agar plate, and the positive clones were picked using p200 pipette tips. The selected plaque was placed in 500 μL Lambda dilution buffer (0.1 M NaCl, 10 mM MgSO_4_, 3.5 mM Tris and 0.01% gelatin), vortexed for 30 s and kept at 4°C. The positive clones underwent secondary immunoscreening with the hyperimmune rat serum and rat-pre-immunisation serum as negative control to exclude false positive clones.

### Sequence analyses

Clones isolated from the cDNA library were amplified by PCR using vector-specific primers and sequenced at the University of Kentucky’s Advanced Genetic Technology Center. The resulting sequences were used as queries in BLASTN searches against non-human, non-mouse nucleotide sequences and BLASTX searches against the non-redundant protein database from NCBI. The presence of a signal peptide was predicted using SignalP 4.0 [39], the presence of glycosylation sites were analysed using the ExPASy Prosite [40] and the presence of transmembrane domains and protein localisation were predicted using TMHMM 2.0 server [41]. Pairwise alignment and phylogenetic comparison was performed using the ClustalOmega software on the EBI-server [42]. Homologues from *Cylicostephanus goldi* (courtesy of Dr. Jane Hodgkinson, University of Liverpool) and *P. equorum* (courtesy of Dr. Georg von Samson-Himmelstjerna, University of Berlin) were obtained and the partial sequences compared by pairwise alignment using the ClustalOmega software.

### Expression of recombinant protein

Primers were designed to amplify the coding sequence from an immune-reactive cDNA clone for subcloning into a pET22b(+) vector. The primer sequences were as follows: Forward: 5’-GATCCATATGCAAAATGGACCTCCACC-3' and reverse: 5'-GATCCTCGAGTCCCTTCATAGCGTCC-3' which incorporated the NdeI and XhoI restriction sites (underlined) to allow for unidirectional cloning. The PCR was performed using Verbatim High Fidelity Polymerase (Thermo Fisher Scientific, Pittsburg, PA, USA) and the amplified fragment was digested with NdeI and XhoI and ligated with the pET22b(+) plasmid. The resulting plasmid was transformed into *E. coli* BL21 cells and expression of the recombinant protein was induced by 1 mM isopropyl-β-D-thiogalactopyranoside (IPTG) at an OD_600nm_ of 0.6 and the cells were incubated under agitation for 9 h at 30°C. The recombinant protein was purified on immobilised cobalt by affinity chromatography using BD TALON resin (Clontech, Mountain View, CA, USA) as per the manufacturer’s protocol for soluble proteins. Purified recombinant protein was stored in aliquots at −20°C.

### Hyperimmune serum against recombinant protein

A total of 300 μg of recombinant protein was resolved in 12% polyacrylamide gels under reducing conditions, a strip of the gel was stained with GelCode Blue stain (Thermo Scientific, Pittsburg, PA, USA) to verify where the recombinant antigen travelled in the gel and the corresponding part of the gel containing the desired molecular size was cut out and shipped to Cocalico Biologicals, Inc. (Reamstown, PA, USA) and used to immunise a guinea pig with Freund’s complete adjuvant for initial immunisation and Freund’s incomplete adjuvant for subsequent boosters. The guinea pig was immunised by 6 inoculations of recombinant antigen over a period of 17 weeks.

### Western blot analyses

#### *S. vulgaris ES*

For Western blot (WB) analysis of anti-adult *S. vulgaris* ES rat serum against both *S. vulgaris* larval and adult ES antigens, the antigens were mixed individually with SDS-PAGE sample buffer containing protein inhibitor cocktail under reducing conditions and resolved in two 1-well 12% polyacrylamide gels. The proteins were transferred to 0.45 μm nitrocellulose membranes by semidry electrophoretic transfer in Tris-glycine buffer. Membranes were blocked for 1 h in PBST (PBS + 0.05% Tween-20), and probed with pre-immunisation rat serum at 1:100 in PBST or hyperimmune rat serum raised against *S. vulgaris* adult ES at 1:100 in PBST. The signal was developed using TMB stabilised substrate for HRP (Promega, Madison, WI, USA).

For WB analysis of anti-recombinant protein guinea pig serum against larval ES, 4.4 μg *S. vulgaris* larval ES was resolved in a 1-well 12% polyacrylamide gels, transferred to a nitrocellulose membrane and blocked. The nitrocellulose membrane was cut and strips were placed in individual trays. The strips were probed with either 500 μL of guinea pig pre-immunisation serum at 1:500 in PBST or guinea pig hyperimmune serum anti-recombinant protein at 1:2,500 in PBST. HRP-conjugated goat anti-horse IgG(T) (Bethyl Laboratories, Inc., Montgomery, TX, USA), were used as secondary antibodies at 1:10,000 in PBST. The signal was developed using Supersignal WestPico chemiluminescent substrate (Pierce, Rockford, IL, USA).

#### Recombinant protein

Recombinant protein (233 ng) was resolved in each of two 1-well 12% polyacrylamide gels by SDS-PAGE under reducing conditions and transferred to nitrocellulose membranes*.* The membranes were blocked and one was placed in a multi-slot apparatus (Bio Rad, Hercules, CA, USA). The blot was probed with 500 μL horse serum samples (1:50 in PBST) in each slot. As secondary antibodies, HRP-conjugated goat anti-horse IgG(T) (Bethyl Laboratories, Inc., Montgomery, TX, USA), were used at 1:10,000 in PBST. The signal was developed using Supersignal WestPico chemiluminescent substrate.

Strips were cut from the other blocked blot and placed in individual trays. The strips were probed with either pre-immunisation guinea pig serum at 1:500 in PBST or anti-recombinant protein guinea pig serum at 1:2500 in PBST. As secondary antibody, HRP-conjugated donkey anti-guinea pig IgG (H+L) (Jackson ImmunoResearch, Inc., West Grove, PA, USA) were used at 1:10,000 in PBST. The signal was developed using Supersignal WestPico chemiluminescent substrate.

### ELISA

The indirect ELISA was optimised by sequential checkerboard titration to the following setup. Individual wells of a 96 well EIA/RIA plate (Costar® easy-wash, Corning Inc., Corning, NY, USA) were coated with 75 μL of recombinant protein diluted to 0.1 μg/mL in PBS and incubated overnight at 4°C. The wells were washed three times for 1 min with PBST and blocked for 1½ h at RT with 200 μL block solution (PBS containing 5% normal goat serum, 1% dry milk powder and 1% Tween-20). The wells were washed once and 75 μL of horse serum diluted 1:50 in diluent solution (block solution in PBST, 1:10) was added to individual wells in duplicates and incubated for 1 h at 37°C. Positive, negative and blank controls were included on each plate in duplicates. The wells were washed five times with PBST, and 75 μL of HRP-conjugated goat anti-horse IgG (H+L) (Jackson ImmunoResearch, Inc. West Grove, PA, USA) diluted 1:10,000 was added to each well and incubated for 1 h at 37°C. The wells were washed five times with PBST and incubated for 10 min at RT in the dark with 75 μL of RT 1-step Ultra TMB ELISA substrate (Thermo Scientific, Rockford, IL, USA) per well. The reactions were stopped with 75 μL 2 M H_2_SO_4_ per well, and the OD_450nm_ determined using an E-max Precision Microplate Reader (Molecular Devices, Sunnyvale, CA, USA) with a photometric range of 0.000 to 4.000 OD and a resolution of 0.001 OD.

#### IgG subclass antibodies

For evaluation of the optimal diagnostic antibody target in serum from 15 horses with known *S. vulgaris* larval infection status, the level of antigen specific IgG subclasses IgGa, IgGb, IgGc and IgG(T) were evaluated using HRP-conjugated goat anti-horse IgGa, IgGb, IgGc and IgG(T) antibodies (Bethyl Laboratories, Inc., Montgomery, TX, USA) at 1:40,000 as secondary antibodies as per the manufacturer’s recommendation alongside the HRP-conjugated goat anti-horse IgG (H+L) secondary antibody as described in the ELISA setup.

After identifying the best antibody target, the secondary antibody dilution for the ELISA was optimised by checkerboard titration.

#### Diagnostic accuracy of ELISA

These ELISAs were performed in June and July, 2012. Serum samples from all horses (*n*=102) and horses seven months and older (*n*=86) were evaluated for the level of antigen specific IgG and IgG(T) antibodies using the optimised ELISA in separate assays. The intra-assay variability of the ELISA was calculated from duplicate measurements from all horses. The inter-assay variability was calculated from the positive and negative controls included in each assay as well as specifically for three horses that were selected on the basis of their rSvSXP-specific IgG(T) OD_450nm_ (OD value given in parentheses). These animals served as a high positive (2.708), an intermediate positive (1.278) and a negative (0.022). From each sample, a volume of 1,000 μL of serum diluted 1:50 in diluent solution was prepared to test triplicate samples on each of four sequential days to evaluate inter-assay variability as a normalised value, percentage of a positive control (PP), as previously described [43].

### Statistical analyses

The statistical program R, version 2.12 [44] was used to generate graphs and perform statistical analyses. For all statistical analyses, a *P*-value less than or equal to 0.05 was considered significant.

#### Evaluation of IgG subclasses

The non-parametric Wilcoxon rank sum test was used to compare IgG levels within each IgG subclass between *S. vulgaris* infected and uninfected horses.

#### Intra- and inter-assay variability

The intra- and inter-assay % coefficient of variability (% CV) was calculated for each series of assays for each of the secondary antibodies. An intra-assay % CV below 10% and an inter-assay % CV below 20% were considered acceptable [45].

#### Receiver operator characteristics (ROC) curve analysis

ROC curve analyses were performed using the software package Epi for R [46] for two sets of horses: all horses (*n*=102) and horses seven months or older (*n*=86). The reason for excluding foals in the second analysis was due to observations of high larval counts and corresponding low PP-values in some of the younger foals. After excluding horses younger than three months of age the remaining horses were all seven months or older. The optimal cut-off was determined on the basis of the ROC curve analysis.

#### Diagnostic accuracy

For the two sets of horses: all horses and horses 7 months and older, the software package EpiR for R [47] was used to calculate the diagnostic accuracy values; sensitivity, specificity, odds ratio, positive likelihood ratio (LR) and negative LR with corresponding 95% confidence intervals.

#### Correlation of arterial S. vulgaris larvae and rSvSXP-specific antibodies

The correlation between the number of *S. vulgaris* larvae in the cranial mesenteric artery and branches and the level of rSvSXP specific IgG(T)-antibodies expressed as the normalised PP-value was evaluated by the Spearman correlation test from the software package fBasics for R [48].

All horses (*n*=102) were categorised by the number of larvae present in the CMA and branches in the following five groups: Group 0: No larvae, migratory tracts or evidence of previous infection (*n*=42); group 1: No larvae, but migratory tracts or evidence of previous infection (*n*=16); group 2: 1–5 larvae (*n*=16); group 3: 6–25 larvae (*n*=17); and group 4: above 25 larvae (range: 25–292) (*n*=11).

A subset of the horses, horses 7 months or older (*n*=86), was categorised by the number of *S. vulgaris* larvae in the CMA and branches in the following five groups: Group 0: No larvae, migratory tracts or evidence of previous infection (*n*=28); group 1: Migratory tracts or evidence of previous infection (*n*=16); group 2: 1–5 larvae (*n*=16); group 3: 6–25 larvae (*n*=17); and group 4: above 25 larvae (range: 25–200) (*n*=9).

The relationship between the different groups and the PP values was evaluated graphically for both sets of horses. A Kruskal-Wallis test was performed to evaluate if there was a significant difference between groups and a multiple comparison test after Kruskal-Wallis performed to identify the significantly different groups using the statistical software package pgirmess for R [42]. This was performed on both sets of horses.

## Results

### Preliminary characterisation of ES and rat anti-ES serum

**Figure 1:**
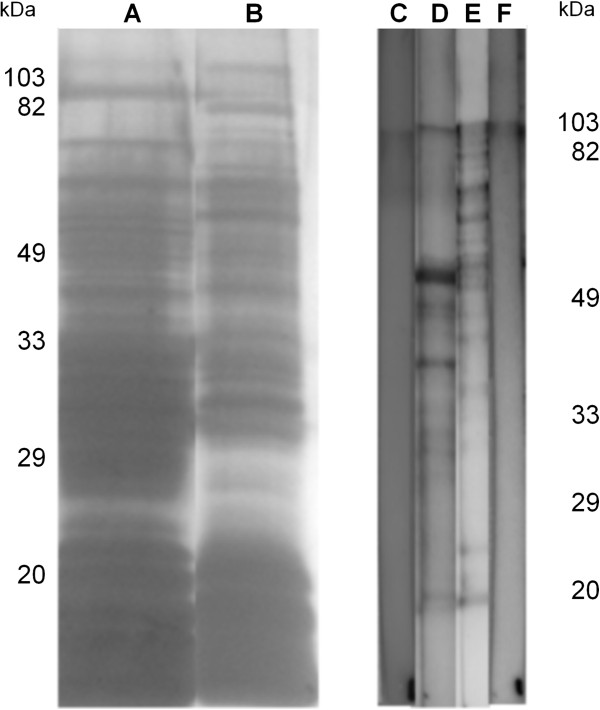
**Silver stained gel.** Lanes A+B illustrate that *Strongylus vulgaris* larval and adult excretory/secretory (ES) fractions contain a highly diverse mixture of molecules. Lane **A**: larval ES, lane **B**: adult ES – both stained by silver staining. To the left the low range molecular markers are shown. Western blot analysis: Lane **C**: reactivity of pre-immunisation rat serum against larval ES; lane **D**: reactivity of anti-adult *S. vulgaris* ES probed against larval ES; lane **E**: anti-adult *S. vulgaris* ES probed against adult ES; and lane **F**: reactivity of anti-adult *S. vulgaris* ES against larval ES. Pre-immunisation serum did not recognise molecules in either ES fraction (lanes **C** and **F**), and the hyperimmune serum recognised more molecules in adult ES (lane **E**) than in larval ES (lane **D**). To the right the low range molecular markers are shown.

The silver staining of the polyacrylamide gel showed a highly diverse mixture of molecules in both *S. vulgaris* larval and adult ES (Figure 1). The hyperimmune rat serum that was raised against *S. vulgaris* adult ES recognised more molecules in the adult ES than it did in the larval ES (Figure 1).

### cDNA library and immunoscreening

Titre analysis indicated that the primary unamplified cDNA library contained 4.75x10^6^ plaque forming units (pfu)/mL. Thirty plaques were randomly selected from the library, and PCR analysis showed that 28 of the clones had inserts (93.3%) and that 26 of the 28 (92.9%) had inserts larger than or equal to 500 bp. Based on BLASTx searches, sequence analysis of the 26 large-insert clones from the quality assessment indicated that they all coded for different gene products (data not shown). Horse serum from naturally infected horses was initially used for immunoscreening of the cDNA library; however, strong levels of anti-*E. coli* antibodies in the horse serum resulted in high background staining that could not be controlled (data not shown).

**Figure 2:**
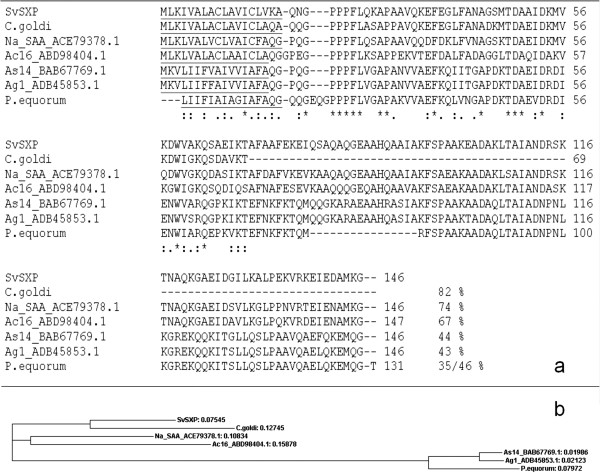
**Sequence comparison by pairwise alignment and a phylogenetic tree. a)** ClustalOmega pairwise alignment of *Strongylus vulgaris* SXP (SvSXP) with homologues in other species. SvSXP (accession number: KC155360) is compared with *Cylicostephanus goldi* SXP partial homologue, *Necator americanus* surface-associated antigen (Na-SAA) (ACE79378.1), *Ancylostoma caninum* immunodominant hypodermal antigen (Ac16) (ABD98404.1), *Ascaris suum* 14 kDa antigen (As14) (BAB67769.1), *Ascaris lumbricoides* antigen 1(Ag1) (ADB45853.1) and *Parascaris equorum* partial homologue. The signal peptide for each sequence is underlined. Asterisks (*) denote identical amino acids, double dots (:) denote conserved amino acid changes and a single dot (.) denotes semi-conserved amino acid changes. Hyphen (−) indicates that a space was inserted in the sequence to improve alignment. The percent similarity to SvSxP is listed for each homologous protein. **b)** Phylogenetic tree of the above mentioned sequences.

The primary immunoscreening of 100,000 clones in the unamplified larval *S. vulgaris* cDNA library yielded 28 positive clones, all of which confirmed positive on secondary immunoscreening. The PCR products of these were all of comparable size (~ 650–680 bp, data not shown) and sequence results of eight slightly different sized clones confirmed that their cDNAs coded for the same protein. The sequence encodes 146 amino acids including a predicted signal peptide (18 amino acids), giving rise to a mature peptide of 128 amino acids with an estimated molecular weight of 13.57 kDa. No glycosylation sites were predicted and the protein did not have any predicted transmembrane domains. The predicted translation showed that the encoded protein is homologous to the SXP/RAL-2 group of nematode specific proteins that contain a conserved Serine-X-Proline motif giving rise to the name. The protein was designated SvSXP. One sequence represented a full-length cDNA coding sequence, which was deposited [GenBank: KC155360]. SvSXP had highest amino acid sequence similarity with a partial sequence from the *Cylicostephanus goldi* homologue, followed by the surface-associated antigen 2 (SAA-2) from *Necator americanus* [Genbank: ACE79378.1], and thirdly the immunodominant-hypodermal antigen AC16 from *Ancylostoma caninum* [Genbank: ABD98404.1]. Two partial sequences from a *P. equorum* derived homologue had a lesser degree of similarity (35% and 46% identities) (Figure 2a). Proteins of SXP-group contain a domain of unknown function (DUF148). The phylogenetic comparison clustered *C. goldi* with *S. vulgaris*, *P. equorum* with *Ascaris suum* and *N. americanus* with *A. caninum* (Figure 2b).

### Analysis of SvSXP in *S. vulgaris* larval ES

**Figure 3:**
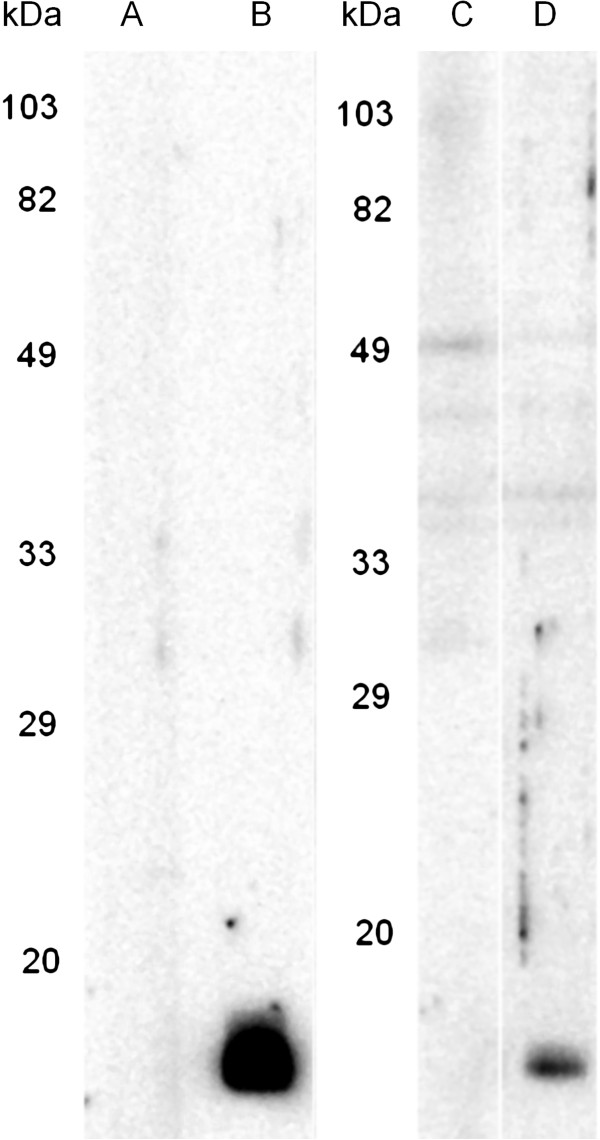
**Western blot analysis of pre-immunisation guinea pig serum (lanes A+C) and hyperimmune anti-rSvSXP guinea pig serum (lanes B+D) against rSvSXP (A + B) and *Strongylus vulgaris *larval excretory/secretory antigens (C + D).** Hyperimmune serum reacted against a similar sized molecule in both antigen mixtures while pre-immunisation serum did not. The molecular weight markers are shown to the left of each type of antigen.

Western blot analyses of rSvSXP and larval ES using hyperimmune guinea pig anti-rSvSXP serum showed that the hyperimmune serum recognised a molecule of similar size in both blots. The pre-immunisation guinea pig serum did not react against the 14 kDa recombinant antigen (Figure 3).

### Western blot analyses of IgG antibodies against rSvSXP in *S. vulgaris*-positive and negative horses

**Figure 4:**
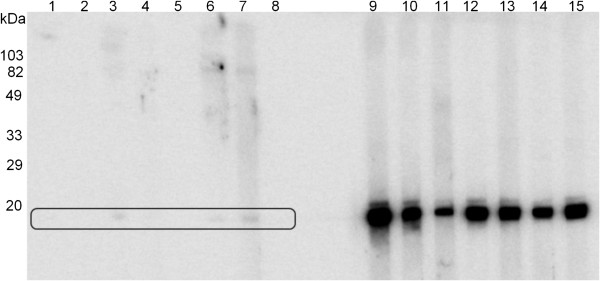
**Western blot with 233 ng recombinant SvSXP.** Lanes 1–8 (box) were probed with serum from *Strongylus vulgaris* negative horse; lanes 9–15 were probed with serum from *S. vulgaris* positive horses. HRP-conjugated goat anti-horse IgG(T) antibodies (Bethyl Laboratories, Inc., Montgomery, TX, USA) were used as secondary antibodies. The molecular weight is indicated to the left.

The immunodiagnostic potential of antigen-specific IgG and IgG(T) antibodies against rSvSXP were evaluated by WB. These analyses revealed no IgG reactivity to rSvSXP in serum from two horses not infected with *S. vulgaris* (data not shown) and low level IgG(T) reactivity to rSvSXP in serum from eight horses not infected with *S. vulgaris* (Figure 4), as determined by post-mortem examination. In contrast, serum from seven horses infected with *S. vulgaris* showed a strong reaction to rSvSXP (Figure 4).

### Diagnostic potential of IgG subclass antibodies

**Figure 5:**
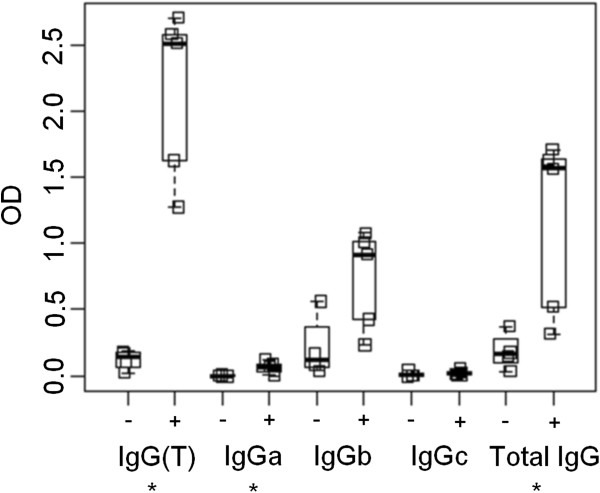
**Boxplot showing optic density (OD) readings of the enzyme-linked immunosorbent assay with antigen specific IgG subclasses grouped by *Strongylus vulgaris *infection status.** -: no *S. vulgaris* larvae; +: *S. vulgaris* larvae present in the cranial mesenteric artery or branches. The middle black line of the box is the median and the range of the box is the inter quartile range (IQR) giving the first and third IQR. The lower and upper lines are up to 1.5 IQR away from the first and third quartile. Each square represents the mean OD_450nm_ reading from one serum sample for each IgG subclass. Asterisks denote significant differences (p<0.05) between uninfected and infected horses within each IgG subclass.

Analysis of rSvSXP-specific IgG subclasses by ELISA showed that detection of rSvSXP-specific IgG(T) antibodies gave the clearest distinction between the seven *S. vulgaris* positive horses and the eight *S. vulgaris* negative horses, while the other IgG subclasses were less consistent (Figure 5). Both the IgG subclasses IgGa (*P*= 0.016*)* and IgG(T) (*P*=0.016) as well as total IgG (*P*=0.032) showed statistically significant differences between infected and non-infected horses (*n*=15), whereas IgGb and IgGc did not.

### Inter- and intra-assay coefficients of variability

The intra-assay variability of the ELISA measuring rSvSXP-specific IgG(T) antibodies was 9.17% for all the duplicate measurements.

For the IgG(T) assays that were included in this study the inter-assay % CV for the positive control was 16.9% while the inter-assay % CV for the negative control was 72.6% resulting in an overall inter-assay % CV of 44.8%. When using the PP-value, the overall inter-assay % CV dropped to 15.6%. The overall inter-assay % CV for the IgG assays, all measured on the same day, was 11.8%.

### Summary of horse material used for evaluation of diagnostic values of the ELISA

The prevalence of arterial lesions as well as the seroprevalence in each group of horses described under Materials and Methods is shown in Table 1.

**Table 1:** **Prevalence of *Strongylus vulgaris *expressed as prevalence of arterial lesions and seropositive horses**

**Group of horses**	**Prevalence of arterial lesions**	**Seropositive prevalence**	**Se**	**Sp**
KY, naturally infected, never treated herd (*n*=11)	100%	90.1%	90.9%	100%
KY, naturally infected, treated herd (*n*=20)	10%	5%	50%	100%
TN, naturally infected (*n*=23)	73.9%	87.0%	94%	33%
TN, experimentally infected with *P. equorum* (*n*=20)	70%	50%	64%	83%
TN, experimentally infected with *S. vulgaris* and Cyathostomins (*n*=28)	57.1%	39.3%	50%	75%

Sensitivity (Se) and specificity (Sp) is presented for each group of horses.

Case definition: A *S. vulgaris* positive horse has presence of larvae, migratory tracts or healing lesions.

Cut-off value used: seropositive: PP-value above 8.75.

A total of 42 horses showed no signs of migrating larvae in the CMA and branches or signs of previous infection and were classified as *S. vulgaris-*negative, larvae with migratory tracts and evidence of thrombosis were found in 47 horses, and migratory tracts or healing lesions were found in 19 horses, of these 10 were determined to show evidence of a previous infection.

**Figure 6:**
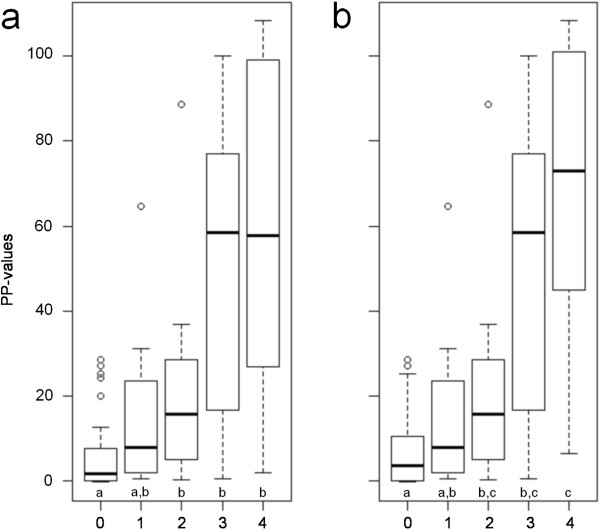
**The relationship between serum anti-rSvSXP IgG(T) antibody levels (expressed as percentage of a positive control (PP)) and counts of migrating *Strongylus vulgaris *larvae categorised by their number of larvae. a**) All 102 horses in the data set. **b**) The 86 horses in the data set aged 7 months and older. Group 0: No larvae, migratory tracts nor evidence of previous infection (Figure 6a: *n*=42; Figure 6b: *n*=28); group 1: Migratory tracts or evidence of previous infection (*n*=16); group 2: 1–5 larvae (*n*=16); group 3: 6–25 larvae (*n*=17); and group 4: >25 larvae (Figure 6a: *n*=11; Figure 6b: *n*=9). The middle black line of the boxes is the median and the range of the box is the inter quartile range (IQR) giving the first and third IQR. The lower and upper lines are up to 1.5 IQR away from the first and third quartile. Groups with different letters are significantly different (*P*-value < 0.05).

In the subset of horses seven months or older, 29 were classified as *S. vulgaris-*negative with no sign of current or previous infection; 42 horses had migrating larvae, migratory tracts and evidence of thrombosis; 17 horses had migratory tracts or healing lesions but no larvae, of these seven were determined to show evidence of a previous infection. The relationship between numbers of migrating arterial *S. vulgaris* larvae and ELISA results is illustrated in Figure 6. In the full dataset there was a significant difference in the level of anti-SvSXP IgG(T) antibodies, expressed as the PP-value, between *S. vulgaris-*negative horses (Group 0) and horses with migrating larvae (groups 2,3 and 4). In the subset of horses seven months and older there was a significant difference between the same groups as well as a significant difference between horses with no larvae but evidence of migratory tracts, aneurysm or healing lesions (group 1), and horses with a heavy infection (group 4).

A moderate positive correlation was found between the number of *S. vulgaris* larvae in the CMA and branches and the PP-value with an R_s_ of 0.5779 (0.433 – 0.693), *P* <0.0001 in the data set including all horses. In the data set including horses 7 months and older there was a moderate positive correlation between the number of *S. vulgaris* larvae in the CMA and branches and the PP-value with an R_s_ of 0.5944 (0.44 – 0.714), *P*<0.0001.

### Diagnostic accuracy

The relationship between sensitivity and specificity is listed for the two datasets at different cut-off values (Table 2).

**Table 2:** **Sensitivity and specificity at different cut-off values of antibody responses against rSvSXP expressed as percent of a positive control (PP), using two sets of horses: A: all horses ( *n *=102); B: horses 7 months or older ( *n *=86)**

**Data set**	**A**		**B**	
PP cut-off	Se^$^	Sp^$^	Se	Sp
5	0.77	0.67	0.78	0.54
10	0.70	0.83	0.71	0.75
15	0.64	0.88	0.66	0.82
20	0.53	0.88	0.55	0.82
25	0.48	0.90	0.50	0.86
30	0.42	0.98	0.43	0.96
40	0.37	1	0.38	1
50	0.32	1	0.33	1
60	0.27	1	0.28	1
70	0.22	1	0.22	1
80	0.17	1	0.17	1
90	0.12	1	0.12	1
100	0.08	1	0.09	1
>100	0.05	1	0.02	1
AUC^$^	0.820		0.783	

^$^: Se: sensitivity; Sp: specificity; AUC: area under ROC curve.

The diagnostic accuracy values expressed as sensitivity, specificity, diagnostic odds ratio, as well as positive and negative LR for each set of horses are shown for the optimal cut-off PP value in Table 3.

**Table 3:** **Diagnostic accuracy values with 95% confidence intervals using the same cut-off value for two sets of horses: A: all horses ( *n *=102); B: horses 7 months or older ( *n *=86)**

**Data set:**	**A**	**B**
Cut-off value (PP)	8.75	13.47
Se	73.3% (60.3 – 83.9%)	65.5% (51.9 – 77.5%)
Sp	81.0% (65.9 – 91.4%)	82.1% (63.1 – 93.9%)
DOR	11.69 (4.48 – 30.5)	8.74 (2.89 – 26.48)
LR+	3.85 (2.03 – 7.32)	3.67 (1.62 – 8.30)
LR-	0.33 (0.21 – 0.51)	0.42 (0.28 – 0.62)

Cut-off values represent optimal sensitivity and specificity for each case-definition. PP: percent of a positive control; Se: sensitivity; Sp: specificity; DOR: diagnostic odds ratio; LR+: positive likelihood ratio; LR-: negative likelihood ratio. The 95% confidence intervals are given in parentheses.

## Discussion

The identification of the *S. vulgaris* SXP protein represents a promising advancement towards development of a diagnostic ELISA test capable of detecting the presence of migrating *S. vulgaris* larvae in horses. In the present study, four important criteria were met by this protein: (i) the recombinant protein was bound by serum IgG(T) primarily from horses harbouring infection or exhibiting lesions associated with previous presence of *S. vulgaris* larvae; (ii) the diagnostic odds ratio of the ELISA showed that infection with migrating *S. vulgaris* larvae significantly increased the possibility of a positive test result; (iii) other naturally occurring equine gastrointestinal nematodes did not appear to interact with test results; (iv) there is evidence of a semi-quantitative relationship between infection intensity and ELISA results.

Similar proteins belonging to the SXP family are expressed in other parasites infecting the horse, including cyathostomins and *P. equorum.* Cyathostomin SXPs appear to be phylogenetically closely related to *S. vulgaris* SXP, and cross-reactivity therefore remains a potential problem for a diagnostic test (Figure 2). Ideally, this could be tested with sera obtained from horses mono-specifically infected with important equine nematodes. Unfortunately, despite many attempts no such sera could be obtained from potential collaborators, and this project did not have the resources to establish such infections. It is still our ambition to establish such infections in the future, and these could be useful to further refine and optimize diagnostic performance of the test. The data generated in this study with horses being either naturally or experimentally infected with major equine helminth parasites. Although, this material illustrates room for improvement of the diagnostic parameters of the ELISA, it also illustrates the diagnostic potential. The experimental infections with *P. equorum* and cyathostomin parasites did not appear to dramatically affect diagnostic performance (Table 1). Further, four out of five of the defined subpopulations returned higher sensitivity than specificity, which would not have been the case if other parasites were stimulating a false ELISA response. It should be borne in mind that sensitivity and specificity for a diagnostic test depends largely on the prevalence of the target organism. Thus, there will always be different values in different study populations. For this reason, it is considered more useful to evaluate the diagnostic odds ratio (DOR) and the likelihood ratios (LR) that are presented in Table 3. The DORs were high and statistically significant, and the test is characterized by a strong positive LR. High levels of cross-reactivity with other species and stages would have led to more false-positive test results, and, thus, a lower positive LR. Taken together, rSvSXP represents a well-characterized and better validated diagnostic antigen for *S. vulgaris* diagnosis than any previously published attempt, and there is good reason to continue developing and refining diagnostic platforms utilizing this protein.

As the hyperimmune rat serum was raised against adult *S. vulgaris* ES antigens and this was used to perform the immunoscreening of the *S. vulgaris* larval cDNA library, the protein must be expressed in both larval and adult stages. Thus, it is possible that adult stages in the intestine are also capable of affecting ELISA measurements. However, previous work with surgically implanted adult *S. vulgaris* worms in the caeca of strongyle-naïve ponies revealed that IgG(T) antibodies in these ponies did not differ from the non-specific background reactivity in strongyle-naïve controls [30]. This suggests that the immune response and hence antibody production is primarily stimulated by the migrating larvae and not the adult worms. Therefore, it remains possible that the presence of the larvae in the blood stream of the horse will allow a higher degree of exposure of the SXP protein to the immune system and, consequently, to a higher degree of production of circulating antibodies against it. More work is needed to investigate this further, but the fair to good relationship found between the number of arterial larvae and ELISA results does suggest that it is primarily the larvae that drive the test results.

Previously, total IgG(T) was associated with the presence of *S. vulgaris* larvae [49] and antigen-specific IgG(T) antibodies have been shown to have immunodiagnostic potential for diagnosing other gastrointestinal helminths in horses [34,50,51]. In the present study, IgG(T) antibodies specific to rSvSXP also appeared to have a better immunodiagnostic potential than other IgG subclasses (Figures 5 and 6). The intra-assay % CV of mean OD_450nm_ values was within acceptable levels whereas the inter-assay % CV was substantially higher. However, the use of the normalised PP value reduced the inter-assay % CV to within acceptable levels, and we therefore recommend reporting results of this assay as PP values.

The SvSXP protein belongs to the SXP/RAL-2 group of proteins that appears to be nematode-specific with several proteins described in animal and plant parasitic nematodes [52-55]. The function of SXP/RAL-2 proteins is currently unknown. The SXP proteins have been shown to be potential vaccine candidates with reduced fecundity of *Ancylostoma caninum* and degree of anaemia in vaccinated dogs [56]. Additionally, SXP-proteins have been used for serodiagnosis of human lymphatic filariasis [53,57] and antibodies against BmSXP-1 and WbSXP-1 were used for detection of circulating filarial antigens [58]. This further supports the possibility that SvSXP possesses the essential qualities required of a diagnostic antigen to be applied for diagnosis of prepatent *S. vulgaris* infection in horses.

Although SvSXP was the only gene identified by immunoscreening the cDNA library, the 26 randomly amplified and sequenced cDNA clones from the quality assessment step were all unique, suggesting that the library is heterogeneous. It seems likely that the SvSXP protein is highly immunogenic so that antiserum raised against ES contains a high proportion of anti-SvSXP antibodies.

The semi-quantitative potential of the test is illustrated in Figure 6. We performed a separate analysis in horses older than seven months to evaluate if the ELISA would perform better in this age group. Indeed, when comparing Figure 6a and b there seems to be a better agreement between the larval burden and the PP-value when foals were excluded. This illustrates that younger foals can harbour a large number of migrating larvae without producing antibody titres, and more studies are needed to explain this finding. However, it should be noted that the AUC of the ROC curves and the diagnostic values calculated from both datasets are not significantly different from each other. Thus, more studies are needed to identify a possible minimum age for the use of the ELISA.

The ELISA performed with a sensitivity and specificity comparable to larval cultures carried out for detection of *S. vulgaris* eggs [59], but has the potential for also reflecting arterial larvae. The odds of a PP-value above the cut-off among horses with migrating *S. vulgaris* larvae, migratory tracts or evidence of previous infection was 11.7 times higher than the odds of a similar ELISA result among horses without migrating *S. vulgaris* larvae or signs thereof. Moreover, a high PP-value indicates a high number of migrating larvae (Figure 6). Even though the sensitivity and specificity are moderate to good, the false negative samples still comprise roughly a fifth of the samples at the optimised cut-off value. For a rare, potentially life-threatening disease the optimal assay would be a highly sensitive test, especially when used at an individual level. This will, however, lead to treatment of 19% of horses with no current infection with *S. vulgaris*. If the test is to function as a screening tool on a herd level it requires a high specificity and the resulting lowered sensitivity in this assay can be counteracted by increasing the number of horses sampled. Table 2 can be used to optimise the interpretation of the test-result on both an individual level and a herd level.

## Conclusion

In conclusion, we have identified a promising candidate antigen for diagnosing *S. vulgaris* infection at the prepatent stage. Our work illustrates that SvSXP is highly immunogenic and that IgG(T) antibodies specific for rSvSXP had the highest diagnostic potential both in WB analysis as well as in an indirect ELISA.

## Competing interests

The University of Kentucky has applied for a provisional patent for the use of rSvSXP and relevant epitopes in the diagnosis of *S. vulgaris*. The inventors, Drs. Martin K. Nielsen, Daniel K. Howe and Ulla V. Andersen, declare to have no conflicts of interest.

## Authors’ contributions

UA collected parasite specimens and serum samples, collected ES antigens, constructed the *S. vulgaris* larval cDNA library, performed quality assurance and immunoscreening of the cDNA library, carried out the sequence analyses, expressed the recombinant antigen, evaluated the antigen in WB and ELISAs, the statistical analyses and provided the initial draft of the paper. DH and SD participated in designing the study, construction of the cDNA library, sequence analyses and revision of the paper. NT participated in statistical analyses and revision of the paper. JM, PN and SO participated in the study design, data interpretation, and revision of the paper. ETL performed a large share of the necropsies, participated in collection of parasite specimens and serum samples from horses, and reviewing the paper. CRR performed experimental infections, necropsies, participated in collection of parasite specimens and serum samples from horses, and revised the paper. MKN conceived and oversaw the study, participated in its design, performed necropsies, collected parasite specimens and serum samples, and revised the manuscript. All authors read and approved the final manuscript.
